# Analysis of the efficacy of two kinds of loss restoration of posterior teeth using 3D printing temporary crown during the second phase of implant surgery

**DOI:** 10.1097/MD.0000000000040460

**Published:** 2024-11-29

**Authors:** Yannan Cao, Meichun Hu, JinLin Zhang, Liuping Yu, Yufeng Gao, Zhuang Ding, Hong Ma, Fangyong Zhu

**Affiliations:** a Department of Stomatology, Affiliated Hospital of Jiangnan University, Wuxi, China; b Wuxi Medical College, Jiangnan University, Wuxi, China; c Wuxi People’s Hospital, Wuxi, China.

**Keywords:** 3D printing technology, A single one, Continuous deletion, Implant restoration, Planting phase II, Temporary crown

## Abstract

**Background:**

This study aimed to analyze the effect of 3D printing temporary crowns on the single and continuous loss restoration of posterior teeth during secondary implant surgery.

**Methods:**

This study adopted a single-center, randomized, single-blind, parallel-controlled trial design. Thirty patients who were admitted to the Department of Stomatology, Affiliated Hospital of Jiangnan University between January 2020 and February 2023 and who had >2 consecutive missing posterior teeth were implanted with the same implant system. A simple random method was adopted to assign 30 patients to group A (using 3D-printed temporary crowns) and group B (control group) in a 1:1 ratio, with 15 patients in each group. The final prosthesis in Experimental group A was done based on the adjustment of the temporary crown and tooth. Both groups were compared based on total time, accuracy, and patient satisfaction of the final prosthesis. Additionally, 30 patients who had undergone single posterior tooth implantation during the same period were selected as the study objects and divided into 2 groups using the random comparison table method. The patients in the experimental group C (15 cases) were provided with 3D-printed temporary crowns, and the other 15 patients were assigned to the control group D (15 cases).

**Results:**

In the experimental group A, the total time spent on dentures was 10.1 ± 1.3 minutes, which was lesser compared with that spent in the control group B (*P* < .05). Experimental group A exhibited a significantly higher accuracy of prosthesis (*P* < .05), as well as significantly higher satisfaction of patients (*P* < .05). Regarding the food impaction rate, after formal implantation and restoration, the food impaction rate of the experimental group C at 1 month and 6 months was compared with that of the traditional 2-stage surgery group D.

**Conclusion:**

Three-dimensional printing temporary crowns exhibited significantly better therapeutic effects for restoration in patients with continuous missing implants in posterior teeth. 3D printing temporary crowns in patients with single posterior tooth implant restoration showed promising results, which improved food immobilization and actively and effectively reduced the complications associated with a single posterior tooth implant.

## 1. Introduction

The restoration of posterior implants is known to be associated with several complications, such as implant rupture, crown loosening, implant fracture, fracture, and screw loosening.^[1–3]^ The traditional methods involving horizontal implant-taking and platform transfer are frequently used in patients with continuous loss of multiple posterior teeth to obtain the final prosthesis after several steps of processing.^[4,5]^ Often, accuracy is affected by errors in the transfer process.^[6,7]^ Also, the repair time, such as grinding beside the chair, is significantly increased. Therefore, there is an urgent need to find ways to improve the accuracy of implant prostheses and reduce the operation time beside the doctor’s chair.^[8,9]^ Previous literature has revealed that although the production process is complicated, a temporary crown could be worn in the mouth with ideal results.^[10–12]^

Food impaction is one of the most common complications involving implant restoration of a single posterior tooth.^[13–15]^ Several studies have investigated the etiology of food impaction,^[16,17]^ especially in implant restoration, such as poor adjacency of dental crown, inadequate marginal ridge, and poor occlusal relationship of the maxillary teeth. The symptomatic treatment, involving the application of personalized healing abutments, jaw tooth adjustment, etc, has also been explored. Food impaction is a dynamic process. The implant abasement plays a vital role as an adjustable factor in the repair process.^[18,19]^ Since the implant depth cannot be changed, food impaction can result from the penetration height of the abasement, the shape of the gingival margin, and the contact surface with the gum cuff. Usually, the available abutments for clinical selection are finished abutments. Since they have limited angles, shapes, and adjustable range and have vast differences in the cylindrical neck shape and the natural neck shape, it is challenging to meet the practical needs of clinical practice.

Here, a 3D-printed temporary crown specially used for the second stage surgery was prepared using the postimplantation CT and the oral data of the original patient after fitting, using Ti-base as the base platform.^[20]^ The study aimed to evaluate the effect of single/continuous missing restoration of posterior teeth and traditional implant restoration on the accuracy of the final restoration and the operation time at the doctor’s chair.

## 2. Data and methods

This study employed a single-center, randomized, single-blind, parallel-controlled trial design. The study protocol was approved by the patients after obtaining their informed consent, and was granted approval by the Medical Ethics Committee of the Affiliated Hospital of Jiangnan University in January 2020 (Approval Number: LS2020059).

### 2.1. Randomization and blinding

This study was approved by the Medical Ethics Committee of the Affiliated Hospital of Jiangnan University in January 2020 (approval number: LS2020059). Informed consent of the patient has been obtained. Thirty patients from the Stomatology Department of the Affiliated Hospital of Jiangnan University due to continuous missing of more than 2 rear teeth between January 2020 and February 2023 were selected. Of these 30 patients, 18 were male and 12 were female, with the youngest being 21 years old and the oldest being 49 years old, with an average age of 32 years. Thirty patients were randomly divided into 2 groups at a 1:1 ratio. Group A (the experimental group) used 3D-printed temporary crowns during the second stage implant surgery, while Group B (the control group) used traditional healing abutments.

In addition, this study also included patients with single non-free posterior tooth implants, and the same random grouping method was adopted. Of these, 10 were male and 5 were female, with the youngest being 25 years old and the oldest being 40 years old, with an average age of 30.35 years. A 3D-printed temporary crown was used in the second phase of implantation in experimental group C, and a conventional healing abutment was used in control group D. There was no significant difference between the demographic data between the 2 groups.

For each patient, a random number was generated, and then these random numbers were sorted. Based on the sorting results, patients who fell in the first half of the sorted list were assigned to the experimental group, while those in the second half were allocated to the control group, ensuring an equal number of patients in each group. The results of this random allocation were recorded. Throughout the course of the study, the patients remained unaware of their group assignments.

### 2.2. Materials and methods

Implant (Zimmer, USA), intraoral scanning rod, post-implant crown material (Wieland Company, Germany), 3D-printed temporary crown (Advance Company, Hangzhou, Zhejiang).

All patients in the experimental group underwent implantation by the same doctor using the implant surgical guide. After healing for >3 months, before the second stage of surgery, the Cone beam Computed Tomography and mouth scan data from patients in the experimental groups A and C was used to prepare the temporary crown Standard Tessellation Language file with Ti-base in 3shape. Before the second surgery, the 3D-printed crown data was formed by fitting the oral scan data used in the postoperative CT of the first surgery and the implant guide made before the first surgery. A 3D printer was used to print the temporary crown. After the temporary crown was generated, it was soaked in 95% ethanol for 5 minutes. After rinsing with normal saline, light curing for 5 minutes and light polishing. This was 3D-printed for clinical use during the second stage of implant surgery, and it facilitated the examination of the adjacency relationship between the prosthesis and neighboring teeth. If the contact tightness, position, and size were found to be incompatible, and the bridge tightness or the occlusal relationship was not optimal, flow resin was used to increase or decrease the relevant parameters. The control groups B and D used the same surgical guide as the experimental group during the second surgery and a circular incision was made in the gum, and the healing base was placed to minimize damage to the gums. One month later, the 3D-printed temporary crown was used as the transfer rod in experimental groups A and C, and the conventional finished transfer rod for platform transfer was used in groups B and D for final restoration. The implants in all 4 groups were fixed using screws. After insertion, the distance between the implant and the adjacent tooth boundary was checked. Also, the physician checked if the dental floss had some resistance. All procedures are performed by the same physician.

### 2.3. Effect evaluation

#### 2.3.1. Group A and group B with continuous loss of posterior teeth

Time of tooth wearing beside the doctor’s chair: the time when tooth wearing started and finished was recorded as the beginning and the end, respectively. The evaluation of the accuracy of the final prosthesis was divided as follows^[21,22]^: (1) Adjacency tightness—floss resistance through, that is, the contact was suitable; if it was easy or difficult to pass, it was considered inappropriate contact; (2) Bridge tightness—if the bridge and gingival fit closely, it was close, else if there was a gap below the bridge, it was not close; (3) Occlusal accuracy—the occlusion was considered appropriate if there was even contact between the posterior implant denture and the natural teeth of the opposite jaw when biting gently, and there was no early contact in the median jaw position, the forward jaw position, and the lateral jaw position; else, the occlusion was inappropriate. 1 implied accurate, 0 implied inaccurate. One week after the restoration was completed, a questionnaire survey was conducted to investigate patient satisfaction, including appearance, chewing function, and comfort level. The total score of each aspect was 100 points; and a score > 70 was considered satisfied, and a score < 70 was considered dissatisfied.

The satisfaction rate was calculated as follows:


$$\begin{array}{l} Satisfaction\;rate=number\;of\;satisf\;ied\;cases/total\;number\;of\;cases\times 100\%. \end{array}$$

#### 2.3.2. Group C and group D with a single posterior tooth missing

① The food impaction rate was recorded by asking patients or based on patients’ complaints about food impaction. ② Mastication efficiency was scored by suspension (peanut) colorimetric method. Different mastication forces between the 2 groups resulted in different degrees of breakage of the peanut and different concentrations of suspension, resulting in variable degrees of corresponding spectral breadth instrument data. ③ Examine whether there were complications such as dental caries, periodontitis, tooth loosening, etc in the adjacent teeth to the dental implants, and compared the incidence rates of these complications between the 2 groups.

### 2.4. Statistical evaluation

The experimental data were statistically analyzed using SPSS19.0 statistical software, and *P* < .05 indicated statistically significant data. The *t* test was used for measurement data, *χ*^2^ test was used for counting data, and α = 0.05 was used as the test level.

## 3. Results

### 3.1. Comparison of tooth wearing time beside chair for restoration of continuous loss of back teeth

Table 1 shows the chair-side operation time and comparison between the 2 groups. There was a significant reduction in the chair-side operation time for experimental group A than that in the control group B.

**Table 1:** Comparison of chairside time between experimental group and control group (*x* ± *s*, min).

Group	n	Temporary crown adjustment	Final restoration adjustment	Total
Experiment group A	15	5.3 ± 1.4	4.8 ± 1.2	10.1 ± 1.3
Control group B	15	0	27.5 ± 10.4	27.5 ± 10.4

### 3.2. Comparison of the accuracy difference of the final prosthesis for continuous posterior tooth loss restoration

Table 2 shows the accuracy of the final restoration of the 2 groups. A significantly higher accuracy was observed in the final restoration system of experimental group A (*P* < .05).

**Table 2:** Comparison of restoration accuracy between experimental group and control group.

Group	n	Touch point (%)		Occlusion (%)		Dummy (%)	
		Accuracy	Inaccuracy	Accuracy	Inaccuracy	Accuracy	Inaccuracy
Experiment group A	15	15 (100)	0 (0)	14 (93.33)	1 (6.67)	13 (86.67)	2 (13.33)
Control group B	15	11 (73.33)	4 (26.67)	8 (53.33)	7 (46.67)	7 (46.67)	8 (53.33)
*χ* ^2^		4.615 .032		6.136 .013		5.400 .020	
*P*							

### 3.3. Difference in satisfaction rate of patients with continuous posterior tooth loss repair

Table 3 shows the patient satisfaction rate in the 2 groups. The patient satisfaction rate was significantly higher in the experimental group A (*P* < .05).

**Table 3:** Comparison of patients’ satisfaction between the experimental group and control group.

Group	n	Appearance (%)		Chew (%)		Comfort level (%)	
		Satisfaction	Dissatisfaction	Satisfaction	Dissatisfaction	Satisfaction	Dissatisfaction
Experiment group A	15	15 (100)	0 (0)	14 (93.33)	1 (6.67)	14 (93.33)	1 (6.67)
Control group B	15	10 (66.67)	5 (33.33)	11 (73.33)	4 (26.67)	10 (66.67)	5 (33.33)
*χ* ^2^		6.000 .014		2.160 .142		3.333 .068	
*P*							

### 3.4. Comparison of food impaction rate after formal restoration of single posterior tooth loss

The food impaction rate was significantly lower in experimental group C compared with that in control group D at 1 and 6 months post-complete implant restoration (*P* < .05) (see Table 4).

**Table 4:** comparison of food impaction scores between the 2 groups after formal repair [n (%)].

Group	n	After formal restoration (month)	
		1	6
Control group D	15	6 (40)	9 (60)
Experiment group C	15	1 (6.67)	3 (20)
*χ* ^2^			
*P*		<.05	<.05

### 3.5. Comparison of chewing efficiency before and after formal restoration of single posterior tooth loss stage II

The masticatory efficiency was insignificantly different between the 2 groups before stage II operation. Post-intervention, there was significantly higher bite force and mastication efficiency of the 3D printing temporary crown in group C than those in the traditional stage II surgery group D (*P* < .01) (see Table 5).

**Table 5:** Comparison of masticatory efficiency between the 2 groups before operation and after formal repair (*x* ± *s*).

Group	Occlusal force		Masticatory performance	
	Before stage II surgery	After formal restoration	Before stage II surgery	After formal restoration
Control group D	88.14 ± 4.92	100.74 ± 8.55	0.43 ± 0.04	0.82 ± 0.15
Experiment group C	88.43 ± 3.15	130.11 ± 8.54	0.41 ± 0.06	1.43 ± 0.11
*t*	‐0.192	‐9.413	1.074	‐12.701
*P*	>.05	<.001	>.05	<.001

### 3.6. Comparison of the incidence of complications of single posterior tooth loss between the 2 groups

Six months post-final restoration, a significantly lower incidence of dental caries, periodontitis, tooth loosening, and other complications was observed in experimental group C than that in the traditional stage II surgery group D (*P* < .05) (see Table 6).

**Table 6:** Comparison of complication rates between the 2 groups.

Group	n	Caries	Tooth mobility	Periodontitis	Abscess	Total
Control group D	15	2	1	2	1	6
Experiment group C	15	0	0	0	0	0
*P*		.483	1.00	.483	1.00	.1

## 4. Discussion

The second phase of implant surgery involves removing the bone and soft tissue that covers the top of the implant. This exposes the implant platform to the mouth. Next, a healing base is placed 3 months after the implant was initially implantation.^[23,24]^ This is one of the primary ways in which gums take shape. Previous studies have used implant surgical guides to place the implants precisely.^[25]^ Using the first-stage surgical guide in the second stage of surgery, the operation time and intraoperative blood loss were substantially reduced, along with the shortening of the healing time of postoperative gingival tissue as well as the pain time, in turn, improving patient satisfaction.^[26]^

Producing an accurate prosthesis plays a critical role in the success of successive implants of multiple posterior teeth. Even a small error during transfer can impact the final product.^[27]^ Zhang Qiang et al^[28]^ used a subjective satisfaction questionnaire and found that patients who received continuous crown repairs had more severe cases of food impaction and crown shedding. In cases where multiple teeth were continuously absent, there was a greater likelihood of occlusal deviation and occlusal strength error. Zhang Ru et al^[29]^ found that occlusal delay could be achieved by leaving a gap of about 30 µm between the implant and the natural teeth of the antijaw, which, in turn, could help reduce force and protect the implant during the initial stage of implant restoration. Kim et al^[30]^ showed that there was a significant increase in the bite force 1-month post-implant restoration, with molar restoration having the highest impact on the bite force. There was an increase in the risk of adjacency loss after implant restoration due to changes in the position and magnitude of bite force.

Single posterior tooth loss constitutes a large proportion of dental implant cases, with multiple complications. Food impaction is commonly observed in clinical practice. Immobility and natural teeth contribute to the formation of food impaction between implanted teeth. Huang Zhibin et al^[31]^ reported a high proportion of food impaction after implant restoration at 84.2%. Another study on the traditional Phase II surgery group in this study supported this claim. At the same time, food impaction can easily cause dental problems, such as dental caries, gingival, periodontal, etc. Food impaction can be categorized into 3 types: vertical type, horizontal type, and mixed type.^[32]^ This study results also confirmed the common observance of level shape caused by alveolar ridge atrophy and soft tissue loss in the missing area, primarily since the conventionally finished implant repair abutment was hard to shape the lost gingival papilla in the missing area.^[33–35]^ In addition, individuals have variable oral conditions. Personalized healing abutments can improve food impact and other complications; however, the production method could be complicated, as described by Pan Wenhui et al.

Here, before the second stage of implantation, a 3D-printed temporary crown was prepared using the postimplantation CT and the oral data of the original patient after fitting, with a Ti base. 3D printing resin temporary crown, through the design software in the computer virtual design of the crown morphological characteristics, high precision in the second phase of tooth wear, easy to adjust. The average tooth-wearing time in experimental group A was 10.1 ± 1.3 minutes during the restoration of multiple posterior missing teeth. Finally, there was a significant reduction in the total time of wearing teeth in experiment group A compared to control group B, saving doctors’ workload. Also, the use of temporary crown repair promoted patients to accurately respond to adjacency, bridge density, occlusal accuracy, and other conditions. Experimental group A of the 3D-printed temporary crown investigated the 3D position and space of the missing area for a second time. The modification of a 3D-printed temporary crown was possible after exposure to common repair problems, such as food impaction and lack of bite prior to the final repair, to readjust it during the final repair.

In group C, maximum restoration of the shape of the gingival papilla and the gingival profile was observed. The soft tissue adaptability benefitted from optimal contact between the upper prosthetic structure of the implant and the remaining natural teeth, which also reduced the degree surrounding dental tissue extrusion by the implant. This method was simple and did not result in an increase in the number of return visits. A significantly higher biting force and chewing efficiency post-intervention was observed in the experimental group C with the 3D-printed temporary crown after formal restoration than in control group D with traditional phase II surgery (*P* < .01).

Regarding patients’ satisfaction rate, a significantly higher satisfaction rate of appearance, mastication, and comfort was observed in experimental group A than in control group B, which was primarily associated with the gum shaping of the temporary crown. The study by Xia Yuning et al confirmed an improvement in patients’ biting force and chewing efficiency due to the insertion of 3D-printed temporary crowns, which was attributed to the personalized healing base to promote gingival papillary recovery and ensure bone mass.^[36]^

Here, the formal repair molds were scanned using intraoral scanning rods, and the data was processed in the machining center. It was ensured the implant was consistent with the patient’s soft tissue. Experimental group C, which used the 3D-printed temporary crown, investigated the 3D position of the missing area again, and the 3D-printed temporary crown was modified after exposure to common repair problems, such as food impaction and lack of bite, prior to the final repair. An optimal contact relationship was achieved with the reserved teeth while fully ensuring the shape of the gum. The final restoration was equivalent to the temporary restoration after reengraving and adjustment. Here, the posterior teeth were implanted using the bone-level Zimmer implant system. The adhesives were not required for the integrated prosthesis made of Ti-base abutment, thus eliminating the side effects caused by adhesives. Also, it promoted the shape of the cuff. The insertion of the 3D-printed temporary crown improved the biting force and chewing efficiency of the patients, which was a part of the personalized healing abutment to recover gingival papillae and ensure bone mass.

Thus, 3D printing has the potential to effectively reduce the working time at the dentist’s chair during implant restoration of continuous loss of posterior teeth with temporary crowns. Also, the completed implant restoration has been shown to be more accurate and to have a higher degree of patient satisfaction. An optimum therapeutic effect was observed in patients with single posterior tooth implantation, which could improve food impaction and actively and effectively reduce the side effects of single posterior tooth implantation, supporting its clinical use. The use of 3D-printed temporary crowns has been widely adopted in clinical practice. However, this study primarily focused on implant restoration in the posterior dental area, which possesses certain limitations. Future research can further expand the sample size and follow-up duration to gain insights into its long-term effects.

## Author contributions

**Project administration:** Meichun Hu.

**Resources:** Hong Ma.

**Supervision:** JinLin Zhang, Zhuang Ding.

**Visualization:** Liuping Yu.

**Validation:** Yufeng Gao.

**Writing – original draft:** Yannan Cao.

**Writing – review & editing:** Fangyong Zhu.
